# Lobomycosis in Soldiers, Colombia

**DOI:** 10.3201/eid2504.181403

**Published:** 2019-04

**Authors:** Claudia M. Arenas, Gerzain Rodriguez-Toro, Andrea Ortiz-Florez, Ingrid Serrato

**Affiliations:** Hospital Militar Central, Bogota, Colombia (C.M. Arenas, I. Serrato); Universidad del la Sabana, Chia, Colombia (G. Rodriguez-Toro);; Fundacion Universitaria Sanitas, Bogota (A. Ortiz-Florez)

**Keywords:** lobomycosis, Jorge Lobo disease, lacaziosis, Lacazia loboi, fungi, fungal infections, leishmaniasis, cutaneous disease, dermatologic analysis, soldiers, military activities, Colombia

## Abstract

Lobomycosis is a disease that is endemic to the Amazon rainforest and is caused by the still uncultured fungus *Lacazia loboi*. This disease occurs in loggers, farmers, miners, fishermen, and persons living near coastal rivers of this region. We report 6 soldiers in Colombia in whom lobomycosis developed after military service in the Amazon area. The patients had nodular and keloid-like lesions on the face, neck, trunk, and limbs. The duration of illness ranged from 2 years to 15 years. The initial diagnosis was leishmaniasis on the basis of clinical manifestations and direct smear results, but biopsies confirmed the final diagnosis of lobomycosis. Treatment with surgical excision, itraconazole and clofazimine was satisfactory. However, the follow-up time was short. Healthcare professionals responsible for the diagnosis and treatment of skin diseases need to be able to recognize the clinical signs of lobomycosis and differentiate them from those of cutaneous leishmaniasis.

Lobomycosis (lacaziosis) is a chronic subcutaneous mycosis caused by the still uncultured fungus *Lacazia loboi* (*1*). Clinical manifestations of this disease are pleomorphic: papules; nodules; and wart-like, ulcerated, and keloid-like lesions located on exposed and cooler areas of the body, particularly the lower limbs and ears (*2*–*7*). Lesions might be single or multiple and are classified in localized or disseminated forms according to their distribution (*7*).

Lobomycosis was first described in 1931 by Jorge Lobo in a 48-year-old man who lived in the Brazilian Amazon and for the previous 19 years had had keloidal nodules in the lumbar region (*8*). Human lobomycosis has been reported in other countries in South America (Brazil, Colombia, Venezuela, and Peru); ≈500 cases have been reported (*2*–*7*). Several names have been used to describe this disease, including lobomycosis, keloidal blastomycosis, Amazonic blastomycosis, Jorge Lobo disease, and lacaziosis. Lacaziosis was named in honor of the Brazil mycologist Carlos da Silva Lacaz, a mycology expert who along with Baruzzi and Rosa published a book that covers all aspects of the disease before 1986 (*2*). This disease affects men in 88% of cases (*7*) likely because it is believed to be related to occupational exposure, especially in forest loggers, rubber tappers, hunters, miners, fishermen, and agricultural workers, as well as residents of the Amazon basin, and indigenous populations in Brazil and Colombia (*2*–*7*,*9*–*12*). In Manaus, Brazil, lacaziosis was the most common fungal infection in the Brazilian Amazon; it accounted for 50 (42%) of 119 cases (*11*). Dolphins are the only animals that have this disease (*13*–*17*). The habitats for *L. loboi* are humid forest areas with temperatures 24°C–32°C, large rivers, and coastal waters. Inoculation of *L. loboi *is through the skin is by trauma; for this reason, lacaziosis is considered a mycosis of implantation (*14*,*17*).

Diagnosis is difficult and often delayed. In a study of 249 cases diagnosed in the state of Acre, Brazil (*7*), diagnoses were delayed by an average of 19 years because the organism grows slowly and produces no major symptoms other than mild pruritus or pain if there is trauma in the lesion. Another reason for delayed diagnosis is that patients do not regularly seek medical services or consult healthcare professionals. *L. loboi* has not been cultured, although it is abundant in lesions. Thus, a diagnosis is made by skin biopsy showing a large number of yeasts of uniform size and thick cell walls that form chains linked by thin bridges that are shown by staining with methenamine silver (*2*,*4*,*9*,*18*). Direct smear and exfoliative cytologic analysis are fast, inexpensive, and accurate diagnostic techniques because they detect abundant numbers of yeast (*19*,*20*). We report 6 soldiers in Colombia who acquired lobomycosis when stationed in a jungle area because of military activities.

## Case-Patients

### Case-Patient 1

**Figure 1:**
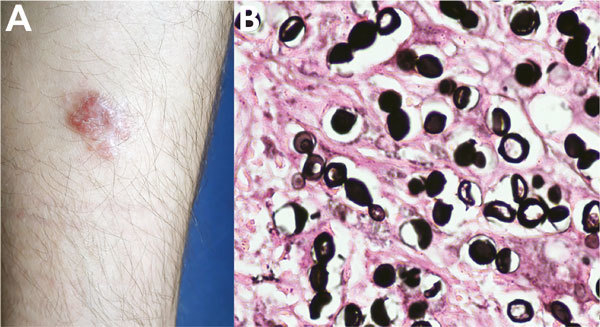
Lobomycosis in a 28-year-old soldier (case-patient 1), Colombia. A) Erythematous papules that became confluent and formed an infiltrated plaque with kelodial aspect, smooth surface, located on the middle third of the right leg. B) Grocott-Gomori staining of a biopsy specimen from the lesion shows chains of yeast, some of them in the budding process. Periodic acid–Schiff stain shows yeast of uniform size with a thick cell wall and clear cytoplasm (original magnification ×40).

A 28-year-old soldier reported a 2-year history of confluent erythematous papules with a smooth surface, which formed a plaque resembling a keloid scar, located on the middle third of the right leg (Figure 1, panel A). These lesions appeared while he was patrolling in the rain forest. A direct smear of the lesion resulted in a diagnosis of cutaneous leishmaniasis. He was treated with N-methyl glucamine for 20 days but showed no improvement.

The patient was later referred to the Central Military Hospital in Bogota, Colombia. A skin biopsy specimen showed dermal granulomatous inflammation with giant cells and histiocytes and numerous rounded, thick-walled mycotic structures, some with budding and formation of chains of <4 cells. Results of staining with periodic acid–Schiff and Gomori methenamine silver were positive for fungi (Figure 1, panel B). The final diagnosis was lobomycosis. Surgical resection of the lesion was performed, and the infection showed no recurrence at 6 months’ follow-up.

### Case-Patient 2

A 41-year-old soldier from a rural area reported a 15-year history of an ulcerated skin lesion in the sternal notch, which he reported was caused by an insect bite. A direct smear for *Leishmania *spp. was reported as showing a positive result. He was treated with N-methyl glucamine for 20 days, and showed complete healing of the lesion. Eight months later, he relapsed and was treated again with N-methyl glucamine for 20 days and showed complete healing. Two months later, he had a keloid plaque in the sternal notch, which was interpreted as a keloid scar that was treated with intralesional triamcinolone acetonide injections; no improvement was observed.

Simultaneously, an acral lentiginous melanoma developed on the second toe of his left foot; an inguinal sentinel node was also positive for melanoma. He was treated by amputation of the first 2 toes and chemotherapy. However, during treatment, the keloid lesion in the sternal notch increased in size. The patient was then referred to the dermatology department of the Central Military Hospital.

**Figure 2:**
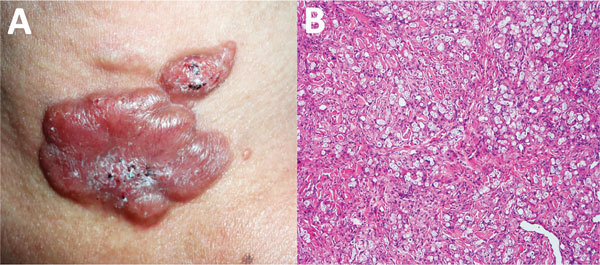
Lobomycosis in a 41-year-old soldier (case-patient 2), Colombia. A) Erythematous, lobulated plaque (4 cm × 2.5 cm) on the sternal notch with hematic crust and black areas on the surface. B) Periodic acid–Schiff staining of a biopsy specimen from the lesion shows the dermis occupied by diffuse, inflammatory granulomata with chains of yeasts (original magnification ×10).

Physical examination showed a lobulated, erythematous plaque (4 cm × 2.5 cm) with a smooth surface with some hematic crust and black areas on the surface (Figure 2, panel A). The suggested diagnoses were metastatic melanoma, lobomycosis, or chromomycosis. A skin biopsy specimen showed typical lobomycosis (Figure 2, panel B). The patient did not receive any treatment for this mycosis; he died as a result of widespread melanoma disease.

### Case-Patient 3

**Figure 3:**
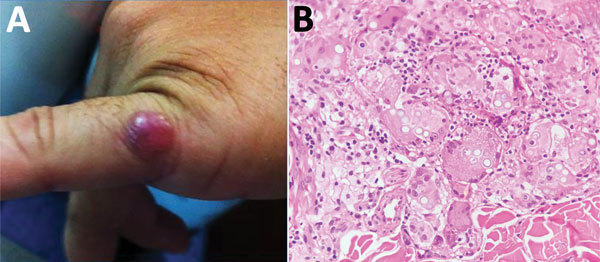
Lobomycosis in a 36-year-old soldier (case-patient 3), Colombia. A) Solitary erythematous-violaceous nodule with a shiny surface and firm consistency (diameter 1 cm) located on the fifth finger of the left hand. B) Hematoxylin and eosin staining of a biopsy specimen from the lesion shows giant cells and numerous yeast structures (original magnification ×20).

A 36-year-old soldier reported a 3-year history of a shiny, erythematous, slow-growing, brown nodule with a smooth surface and firm consistency (diameter 1 cm) on the fifth finger of the left hand (Figure 3, panel A). He had no history of trauma and was asymptomatic. A skin biopsy specimen showed typical features of lobomycosis (Figure 3, panel B). Surgical excision was performed, and he was prescribed oral itraconazole (100 mg /d) for 3 months. There was no recurrence of the lesion after a 1-year follow-up.

### Case-Patient 4

**Figure 4:**
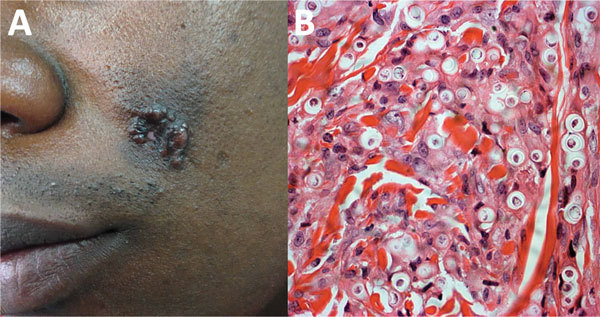
Lobomycosis in a 30-year-old soldier (case-patient 4), Colombia. A) Phototype V lesion on the left cheek with papules that became confluent and formed a lobulated plaque with a smooth and shiny surface. B) Periodic acid–Schiff staining of a biopsy specimen from the lesion shows multiple yeasts of uniform size, and thick walls are seen inside phagocytoses (original magnification ×40).

A 30-year-old soldier reported a 3-year history of an asymptomatic, euchromic, lobed plaque with a smooth and shiny surface on the left cheek (Figure 4, panel A). Slow growth of this lesion became apparent after an insect bite. A direct smear was positive for *Leishmania* spp., and he was given N-methyl glucamine for 20 days. He showed no improvement and was given a second cycle of this drug. A skin biopsy specimen indicated lobomycosis (Figure 4, panel B).

Surgery for the lesion was performed, and the patient was prescribed itraconazole (100 mg/d) for 6 months and clofazimine (50 mg/d) for 6 months. After a 6-month follow-up, he had a recurrence of the lesion. A wide local excision was performed, and he was prescribed oral itraconazole (100 mg/d) for 3 months. He showed no recurrence of the lesion after a 6-month follow-up.

### Case-Patient 5

**Figure 5:**
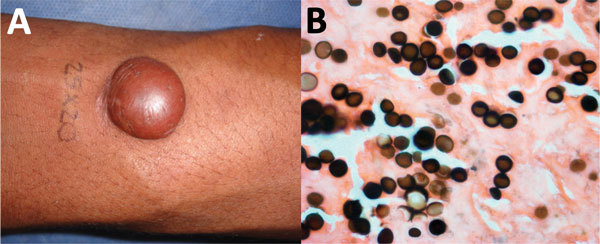
Lobomycosis in a 32-year-old soldier (case-patient 5), Colombia. A) Solitary yeast erythematous nodule (4 cm × 3.5 cm) resembling a keloid scar with a smooth and shiny surface on the right arm. B) Grocott staining of a biopsy specimen from the lesion shows typical chains (original magnification ×40).

A 32-year-old soldier reported a 5-year history of a euchromic nodule (4 cm × 3.5 cm) resembling a keloid scar with a smooth and shiny surface and progressive growth on the right arm (Figure 5, panel A). The patient had no history of trauma and was asymptomatic.

Microscopic findings and staining with periodic acid–Schiff and Gomory methenamine silver showed a diffuse dermal infiltrate of macrophages containing *L. loboi* (Figure 5, panel B). Complete excision was performed. He showed no recurrence of the lesion after a 3-month follow-up.

### Case-Patient 6

**Figure 6:**
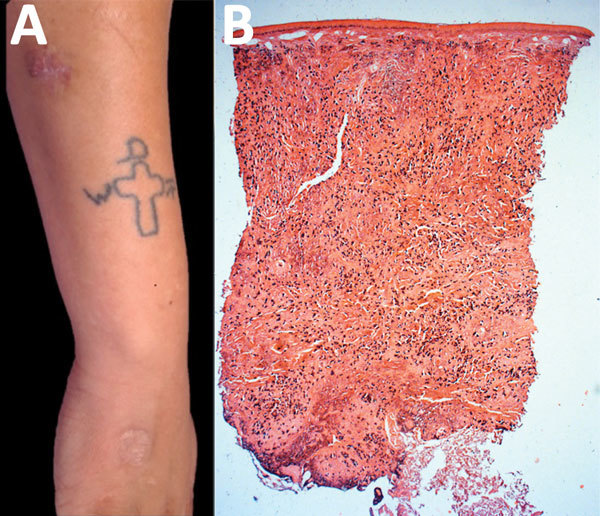
Lobomycosis in a 24-year-old soldier (case-patient 6), Colombia. A) Erythematous-violaceous infiltrated plaque (4 cm x 3 cm) with a shiny surface on the right forearm and typical leishmaniasis cicatricial plaque on the same limb. B) Testing of biopsy sample from the lesion. Epidermis shows no hyperplasia or ulceration, but dermis shows diffuse inflammation rich in vacuolated macrophages; Grocott-Gomori staining shows yeast cells (original magnification ×2.5).

A 24-year-old soldier reported a 2-year history of a keloid-like asymptomatic lesion that slowly increased in size on the right forearm. Physical examination showed an infiltrated, erythematous, violaceous, nonpainful plaque (4 cm × 3 cm) with a shiny surface (Figure 6, panel A). He had a history of cutaneous leishmaniasis in the right hand and had been treated with N-methyl glucamine for 20 days. After seeing no improvement, a second treatment cycle was prescribed.

The patient showed resolution and formation of a scar 4 years later. Histologic analysis showed macrophages and giant cells, numerous yeast structures of uniform size and thickness, refringence of the cell wall, and chains resembling rosary beads. Staining with periodic acid–Schiff and Grocott was positive for fungi (Figure 6, panel B). Surgical resection was performed, and remission of the lesion was observed at a 6-month follow-up.

## Discussion

In Colombia, lobomycosis occurs in Amoruas and Motilones aboriginal communities in the Amazon and Orinoco regions and in black persons in the Pacific Coast regions of Cauca and Choco (*3*,*9*,*21*,*22*). The presence of this disease in soldiers from Colombia who are stationed in jungle or forest regions is a unique situation in which all factors (tropical climate, temperature, humidity, rainfall) related to an optimal habitat for the fungus are present (*2*–*7*,*17*,*21*,*22*).

Lobomycosis is found in Central and South America (Mexico, Costa Rica, Panama, Venezuela, Colombia, Guyana, Bolivia, Suriname, Ecuador, and Peru) (*2*–*7*). Cases that develop outside the Amazon region need an ecology similar to that of jungle areas (*3*–*7*,*23*). Rare cases have been reported in North America and Europe in persons who have traveled to countries in South America (*24*–*28*). However, 2 cases have occurred in persons outside Central and South America who had no history of travel: 1 in a young man who reported swimming in South Africa (*29*) and 1 in a farmer (woman) who lived in a river basin in Greece (*30*).

The natural reservoir of *L. loboi* is believed to be aquatic or associated with soil and vegetation. Transmission of this fungus occurs by skin trauma after incubation periods of months to decades. The initial lesion is a papule at the site of local trauma that typically progresses slowly and shows different clinical manifestations. It has also been suggested that inoculation of the fungus into tissues can occur through insect bites, as reported by 2 of the soldiers in our study, or by snake bite trauma or helix trauma caused by a fish bone (*2*,*6*–*9*,*13*,*17*).

The most common manifestation of lobomycosis is a nodular keloid appearance with slow insidious onset. Case-patients also have macules, plaques, and infiltrative lesions, and as the disease progresses, lesions become verrucous and can ulcerate secondary to trauma (*2*–*13*). The lesion might remain localized, single or multiple, confluent in the same region, or can disseminate and become diffuse over several areas of the body (*2*–*13*). The 6 soldiers reported localized, circumscribed, nodular, and keloid lesions and chronic evolution, which are the most usual manifestations in 60% of case-patients. Case-patient 4 had a lesion on the face, which is present in only 7% of case-patients, and case-patient 2 had a lesion on the neck, which is present in only 1% of case-patients (*7*,*10*,*12*,*31*).

The time of evolution for the lesions of the 6 soldiers ranged from 2 to 15 years, which confirms the chronicity of this infection. Other reported case-patients have had evolution times of 35 to 64 years (*7*,*17*,*31*,*32*). Regional lymph nodes can be affected, but systemic dissemination is rare. Progression of lobomycosis might lead to deformities affecting quality of life and decreasing work productivity of case-patients (*17*).

Impaired cellular immune responses cause chronicity of lesions and facilitate the abundance of fungus inside lesions (30%–50% of fungal cells are viable) (*33*). Macrophages infiltrated by *L. loboi *have increased levels of transforming growth factor-β (an interleukin that inhibits expression γ-interferon and nitric oxide), which suppresses the lytic activity of macrophages and promotes fibrosis production, which contributes to the keloid appearance of lesions (*34*). The inflammatory infiltrate has few lymphocytes and contains neutrophils that are active against the fungus (*35*). There are no plasmacytoid dendritic cells to promote the Th1 response for antigen presentation and migration to lymph nodes (*36*). Blood and lymph vessels are decreased in lesions (*37*). Nodular lesions have cellular components that result in more active immune response cells and less active cells in infiltrative keloids and wart-like and disseminated lesions (*38*).

Three of the soldiers received diagnoses of leishmaniasis after direct observations of specimens by microscopy. These patients received antileishmanial treatment that resulted in healing for 1 of the patients, suggesting that this patient actually had leishmaniasis. Thus, *L. loboi* could have been implanted on the scar caused by leishmaniasis. A similar condition occurred in a patient in Brazil (*31*). Lobomycosis has been associated in the same patient with other diseases, such as ringworm, leprosy, paracoccidioidomycosis, chromomycosis, and leishmaniasis (*7*,*13*,*31*), as in case-patient 6 in our study.

Identification of amastigotes in the direct smear for 3 of these soldiers can also be attributed to common geographic areas for leishmaniasis and lobomycosis. During 2004–2012, >50,000 cases of leishmaniasis were reported worldwide (*39*), many of which were related to combat and destruction of illicit crops. Another reason for detection of lobomycosis and leishmaniasis in the same patient is that health professionals performing cytologic analysis in rural areas do not have enough experience identifying of agents other than *Leishmania *spp.

The biopsy specimens of the 6 case-patients confirmed the diagnosis; these specimens showed granulomatous inflammation without abscesses and large numbers of phagocytized yeast of uniform size that formed chains linked by tiny and thin bridges (*3*,*18*,*40*). Once these manifestations are observed, the diagnosis of lobomycosis should not be confused with other diseases. Molecular taxonomy was used to identify an imported case of lobomycosis in a traveler from Europe who visited the Amazon region of Venezuela (*41*). However, in disease-endemic countries, the diagnosis should include clinical and microscopic results because molecular diagnosis might not be available (*42**,**43*)

Lobomycosis can be confused with other cutaneous diseases. Other diagnoses include cutaneous leishmaniasis, as occurred for 2 of the soldiers, as well as diffuse leishmaniasis, chromomycosis, sporotrichosis, lepromatous or tuberculoid dimorphic leprosy, mycetoma, phaeohyphomycosis, pyoderma, Kaposi sarcoma, sarcoidosis, keloid scars, histiocytosis of Langerhans cells, melanoma, dermatofibrosarcoma, lymphoma, squamous cell carcinoma, and cutaneous metastases (*3*,*17*,*40**,**41*). Thus, a skin biopsy is necessary to confirm the diagnosis.

Cutaneous leishmaniasis is the most common condition in the differential diagnosis for lobomycosis, especially if the lobomycosis is ulcerated, which is a frequent complication. Four case-patients with lobomycosis, 1 of whom died, were reported to have squamous cell carcinomas (*3*,*44*). However, the acrolentiginous melanoma that resulted in the death of case-patient 2 in our report was not related to lobomycosis. Lobomycosis has been reported to occasionally affect the regional lymph nodes (*3*,*45*). However, even when disseminated, cutaneous forms do not affect the mucosa or internal organs. Rare spread of lobomycosis to the testis was reported in a case-patient in Costa Rica (*3*).

Although surgical excision with wide margins is the most recommended treatment for isolated lobomycosis lesions, relapses still occur, as for case-patient 4. Electrocautery and cryosurgery are alternative options. However, there are no effective antimycotic drugs for lobomycosis (*17*,*46*). 

In Manaus, Brazil, where lobomycosis has been the most common deep mycosis, clofazimine (200 mg/d for 13 months) resulted in improvement in 22 patients (*31*). This drug might have contributed to healing of leprosy lesions and concomitant lobomycosis for patients in Acre, Brazil, who had completed multidrug therapy for leprosy (*7*). Rifampin has also shown effective antimycotic activity (*17*). Itraconazole (100 mg d) and clofazimine (100 mg/d) resulted in healing of an extensive plaque on the face of a woman, who did have recurrent clinical or histological lesions after 3 years of follow-up (*47*).

We have used itraconazole and surgery treatment for other patients and have observed good results, although the follow-up time should be years. One of the patients we studied showed recurrence of a lesion 10 years after it was removed (*30*), and a woman who showed remission of a plaque on her face after treatment with itraconazole and clofazimine showed recurrence after 8 years of apparent cure (*11*). Posaconazole (400 mg 2×/d for 27 months) resulted in curing of a patient with lobomycosis in Peru who had a long-lasting lesion (27 years) in the ear (*47*). In addition, analysis of several genomic DNA sequences and of molecular data showed that cutaneous granulomas in dolphins were caused by a novel *Paracoccidiodes* species (*48*,*49*). Those results suggest that a novel, uncultivated strain of *P. brasiliensis* restricted to cutaneous lesions is probably the cause of lacaziosis/lobomycosis in dolphins; however, more research is needed for confirmation.

In conclusion, we report 6 soldiers of the National Army of Colombia who had localized lobomycosis with nodular and keloid-like lesions, acquired while on duty in the Amazonian forests of eastern Colombia in the departments of Caqueta, Meta, and Guaviare, and a jungle area in Montes de Maria in​​ the department of Bolivar. These findings indicate a new and unique epidemiologic situation. Clinical diagnoses and direct smear results were suggestive of cutaneous leishmaniasis for 3 of these case-patients. However, biopsy led to an accurate diagnosis of lobomycosis. Surgery was successful treatment for 3 of the case-patients. Health personnel of the Armed Forces of Colombia, including physicians, bacteriologists, and pathologists, should become better acquainted with this disease to improve its diagnosis and treatment.
